# A predictive score for optimal cytoreduction at interval debulking surgery in epithelial ovarian cancer: a two- centers experience

**DOI:** 10.1186/s13048-018-0415-y

**Published:** 2018-05-30

**Authors:** Eleonora Ghisoni, Dionyssios Katsaros, Furio Maggiorotto, Massimo Aglietta, Marco Vaira, Michele De Simone, Gloria Mittica, Gaia Giannone, Manuela Robella, Sofia Genta, Fabiola Lucchino, Francesco Marocco, Fulvio Borella, Giorgio Valabrega, Riccardo Ponzone

**Affiliations:** 1 Candiolo Cancer Institute FPO-IRCCS, Strada Provinciale 142 km 3.95, 10060 Candiolo, TO Italy; 2 Department of Oncology, University of Torino, Turin, Italy; 3 Department of Surgical Sciences, Gynecology, AOU, Città della Salute e della Scienza, Turin, Italy

**Keywords:** Ovarian cancer, Interval debulking surgery, Optimal cytoreduction, Predictive score, Peritoneal cancer index

## Abstract

**Background:**

Optimal cytoreduction (macroscopic Residual Tumor, RT = 0) is the best survival predictor factor in epithelial ovarian cancer (EOC). It doesn’t exist a consolidated criteria to predict optimal surgical resection at interval debulking surgery (IDS). The aim of this study is to develop a predictive model of complete cytoreduction at IDS.

**Methods:**

We, retrospectively, analyzed 93 out of 432 patients, with advanced EOC, underwent neoadjuvant chemotherapy (NACT) and IDS from January 2010 to December 2016 in two referral cancer centers. The correlation between clinical-pathological variables and residual disease at IDS has been investigated with univariate and multivariate analysis. A predictive score of cytoreduction (PSC) has been created by combining all significant variables. The performance of each single variable and PSC has been reported and the correlation of all significant variables with progression free survival (PFS) has been assessed.

**Results:**

At IDS, 65 patients (69,8%) had complete cytoreduction with no residual disease (*R* = 0). Three criteria independently predicted *R* > 0: age ≥ 60 years (*p* = 0.014), CA-125 before NACT > 550 UI/dl (*p* = 0.044), and Peritoneal Cancer Index (PCI) > 16 (*p* < 0.001). A PSC ≥ 3 has been associated with a better accuracy (85,8%), limiting the number of incomplete surgeries to 16,5%. Moreover, a PCI > 16, a PSC ≥ 3 and the presence of R > 0 after IDS were all significantly associated with shorter PFS (*p* < 0.001, *p* < 0.001 and *p* = 0.004 respectively).

**Conclusions:**

Our PSC predicts, in a large number of patients, complete cytoreduction at IDS, limiting the rate of futile extensive surgeries in case of presence of residual tumor (R > 0). The PSC should be prospectively validated in a larger series of EOC patients undergoing NACT-IDS.

## Background

Ovarian cancer is the leading cause of death from gynecological malignancies. In 2017, 22,400 new cases are expected in the United States. Currently, more than 75% of women with ovarian cancer have advanced disease [International Federation of Gynecology and Obstetrics (FIGO) stage IIIC or IV] at diagnosis and their 5-years survival rate is less than 30% [1].

Primary debulking surgery (PDS) followed by platinum-based chemotherapy has long been considered the only standard treatment for advanced epithelial ovarian cancer (EOC) [2]. This approach validity has been supported by several retrospective studies consistently demonstrating that upfront optimal cytoreduction (residual tumor nodules ≤1 cm or *R* ≤ 1) is associated with longer survival [3, 4]. Unfortunately, PDS is not always associated with optimal cytoreduction and can be complicated by severe perioperative morbidity [5, 6]. More recently, neoadjuvant chemotherapy (NACT) with delayed surgery (interval debulking surgery, IDS) is increasingly adopted in patients with advanced EOC [7]. This tendency is sustained by the results of two randomized phase III trials, showing that NACT-IDS improves optimal debulking rates and reduces surgery-related complications with no detrimental effect on survival, in comparison with PDS, at least in patients with high tumor load [8, 9]. However both trials have been criticized for the poor performances of PDS arm [10, 11].

However, a significant proportion of patients cannot be optimally cytoreduced even after NACT-IDS and this leads to the morbidity of surgery with no expected survival benefit [12–14]. Although it is common practice to attempt IDS only in patients responding to NACT, this approach causes several unnecessary laparotomies, if optimal cytoreduction cannot be achieved, and in other cases they are not applied also if the conditions are appropriate. Single variables have been combined into predictive cytoreduction models to improve accuracy in the settings of PDS [15] and recurrent disease [16, 17]. Unfortunately, predictive models have not been developed for patients undergoing IDS.

Therefore, the aim of this study is to develop a predictive model of surgical outcome at IDS, to improve the selection of patients that can benefit of a maximal surgical effort.

## Methods

### Study population

A total of 432 patients with histologically confirmed diagnosis EOC have been operated between January 1st, 2010, and December 31st, 2016 at Candiolo Cancer Institute-IRCCS and Sant’Anna Hospital, two high-volume gynecological cancer centers in the North-West of Italy. All patients had preoperative computed tomography (CT) of the chest, abdomen and pelvis with intravenous contrast and serum Ca-125 assessment. In all cases, a multidisciplinary board, including a gynecologist and/or a surgeon, a medical oncologist and a radiologist with specific training and expertise in ovarian cancer evaluated the feasibility of surgical resection. The patients underwent PDS when optimal cytoreduction has been deemed achievable, while NACT - IDS was the preferred option when the extent/localization of the disease would likely preclude optimal cytoreduction and/or the patient would not tolerate extensive surgery due to age or co-morbidities. All 93 patients who underwent both NACT and IDS were included in the present study. The following variables has been prospectively entered into a database and retrospectively analyzed: age, performance status (PS) according to Eastern Cooperative Oncology Group (ECOG), comorbidities according to the Chronic Disease Score (CDS) [18], FIGO stage, grade and histology, serum CA-125 at diagnosis before surgery and after IDS [19], type of chemotherapy, peritoneal cancer index (PCI) according to Sugarbaker [20] at IDS assessed during laparoscopy, residual disease (R) after IDS, date of radiological progression (PD) after chemotherapy or last follow-up.

All patients signed a written informed consent and the institutional review board of our Institutions provided their approval.

### Statistical analysis

We performed univariate and multivariate logistic regression analysis, Fisher exact test and chi-square test to search patients’ and tumors’ characteristics that were predictive of complete cytoreduction. Receiver Operating Curve (ROC) analysis has been also adopted to assess the best cut-off values to predict the likelihood of incomplete cytoreduction at IDS of continuous variables. We used all significant variables at multivariate analysis to create a predictive score of cytoreduction (PSC). We assigned one or two points to each criterion, according to accuracy (1 point if < 75%, 2 points if ≥75%). We estimated progression-free survival (PFS) with the Kaplan-Meier method and we compared it using the log-rank test. We considered *p* < 0.05 statistically significant. We performed all analyses using the SPSS statistical software program, version 22.0 (IBM SPSS Inc., Chicago, IL, United States of America).

## Results

Ninety-three patients with predominantly advanced stage (FIGO IIIC-IV: 75,3%), serous high grade (87%) EOC undergoing NACT and IDS were enrolled. At the time of diagnosis, median CA-125 was 2121 UI/dL (range 28–10,454 UI/dL) and Chronic Disease Score (CDS) was ≥2 in 34,4% of the patients. Carboplatin plus paclitaxel was the most utilized chemotherapeutic regimen (87,3%), with only three patients receiving carboplatin single-agent and two patients receiving carboplatin plus pegylated lyposomal doxorubicin, due to hypersensitivity to paclitaxel. Sixty-five patients (69,8%) had complete cytoreduction at IDS. For continuous variables, ROC analysis identified age ≥ 60 years, CA-125 levels before NACT > 550 UI/dL, CA-125 levels after NACT > 33 UI/dL, CA-125 reduction after NACT < 96% and PCI > 16 as optimal cut-offs to predict the surgical outcome. All the above mentioned variables were significantly correlated with incomplete cytoreduction at univariate analysis. However, at multivariate analysis, only age (*p* = 0.007), CA-125 before NACT (*p* = 0.014) and PCI (*p* < 0.001) maintained the statistical significance. For complete baseline patients’ characteristics and statistical correlations see Table 1.

**Table 1:** Univariate and multivariate analysis of variables associated with incomplete cytoreduction at interval debulking surgery

	Total (93Pts.)	R0 (65Pts.)	Non-R0 (28 Pts.)	*Uni-variate p value*	*Multi-variate p value*
Age, years					
Median (range)	60 (36–82)	59,5 (36–82)	65,7 (47–82)	NS	
Age ≥ 60	54 (58%)	32 (49,2%)	22 (78,6%)	0.011	0.007
FIGO stage					
IIIA	9 (9,7%)	6 (9,2%)	3 (10,7%)		
IIIB	14 (15%)	9 (13,8%)	5 (17,9%)	NS	
IIIC	58 (62,4%)	43 (66,2%)	15 (53,5%)		
IV	12 (12,9%)	7 (10,8%)	5 (17,9%)		
Histology					
High-grade serous	81(87%)	57 (87,6%)	24 (85,7%)		
Endometroid	4 (4,3%)	2 (3,1%)	2(7,1%)	NS	
Mucinous	2 (2,2%)	1 (1,5%)	1 (3,6%)		
Clear cell	2 (2,2%)	2 (3,1%)	0		
Other/non specified	4 (4,3%)	3 (4,6%)	1 (3,6%)		
ECOG Performance Status					
0	34 (37%)	26 (40%)	8 (29,6%)	NS	
1	44 (47,8%)	30 (46,2%)	14 (51,9%)		
2	15 (15,2%)	9 (13,8%)	6 (21,4%)		
Ca 125 values, UI/dl					
Median CA-125 at diagnosis (range)	2121 (10454–28)	1964	2793	NS	NS
CA-125 at diagnosis > 550	71 (76,3%)	46 (70,8%)	25 (89,3%)	0.044	0.014
Median CA-125 post NACT (range)	342 (2620–7)	163	598	0.055	NS
Ca 125 post NACT > 33	60 (65,9%)	35 (55,6%)	25 (89,3%)	0.002	NS
CA 125 reduction post NACT < 96%	34 (38,2%)	26 (41,9%)	8 (29,6%)	0.034	NS
Chronic Disease Score (CDS)					
1	61 (65,6%)	44 (67,7%)	17 (60,7%)		
2	24 (25,8%)	17 (26,2%)	7 (25%)	NS	
3	8 (8,6%)	4 (6,2%)	4 (14,3%)		
Peritoneal Cancer Index					
0–16	68 (73,1%)	58 (85,3%)	10 (35,8%)	< 0.001	< 0.001
> 16	25 (26,9%)	7 (10,7%)	18 (64,2%)		
Chemotherapy regimen					
- Carboplatin plus paclitaxel	81 (87,3%)	57 (87,6%)	24 (85,7%)	NS	
- Single agent carboplatin	3 (3,2%)	2 (3,1%)	1 (3,6%)		
- Carboplatin plus PLD	2(2,2%)	2 (3,1%)	0		
- Carboplatin plus paclitaxel plus bevacizumab	7 (7,6%)	4 (6,2%)	3 (10,7%)		

*Pts* patients, *R0* complete cytoreduction, *FIGO* International Federation of Gynaecology and Obstretics, *ECOG* Eastern Cooperative Oncology Group, *NACT* neoadjuvant chemotherapy, *PLD* pegylated liposomal doxorubicin, *NS* not significant

PCI was the best predictor of surgical outcome, with accuracy more than 80% (Table 2). Therefore, we modeled a predictive score of incomplete cytoreduction (PSC) by assigning a value of 1 point to age and CA-125 at diagnosis and 2 points to PCI according to accuracy.

**Table 2:** Diagnostic performance and assigned score of significant variables of incomplete cytoreduction at interval debulking surgery

Variable	Sens (%)	Spec (%)	NPV (%)	PPV (%)	Acc (%)	Assigned score^a^
Age > 60 years	78,6	50,7	84,6	40,7	59,1	1
CA-125 at diagnosis ≥550 UI/dl	89,2	29,2	86,3	35,2	47,3	1
PCI > 16	62,5	90,1	85,9	71,4	82,3	2

^a^To develop a predictive score of cytoreduction (PSC) for each criterion 1 point was assigned if accuracy is < 75% and 2 points if > 75%. *SE* sensitivity, *SP* specificity, *NPV* negative predictive value, *PPV* positive predictive value, *Acc* accuracy

If applied, a PSC ≥3 could have selected all patients for whom complete cytoreduction was not achievable (100%) by limiting at 16,5% the rate of surgical attempts leading to a *R* > 1 cm (Table 3).

**Table 3:** Diagnostic performance of significant variables of incomplete cytoreduction at interval debulking surgery

Variable	NPV (%)	Unnecessarily explored (1-NPV) (%)	PPV (%)	Inappropriately unexplored (1-PPV) (%)
Age > 60 years	84,6	15,4	40,7	59,1
CA-125 at diagnosis > 550 UI/dl	86,3	13.7	35,2	64,6
PCI > 16	85,9	14,1	71,4	28,6
PSC > 3	83,5	16,5	100	0

*CA-125* Cancer Antigen 125, *PCI* Peritoneal Cancer Index, *PSC* Predictive score of cytoreduction, *NPV* negative predictive value, *PPV* positive predictive value, *Acc* accuracy, Unnecessary explored (1-NPV): number of cases that would be considered as resectable disease but non-optimally cytoreduced at laparotomy; Inappropriately unexplored (1-PPV): number of cases that would be considered as unresectable but optimally cytoreduced after laparotomy

**Fig. 1:**
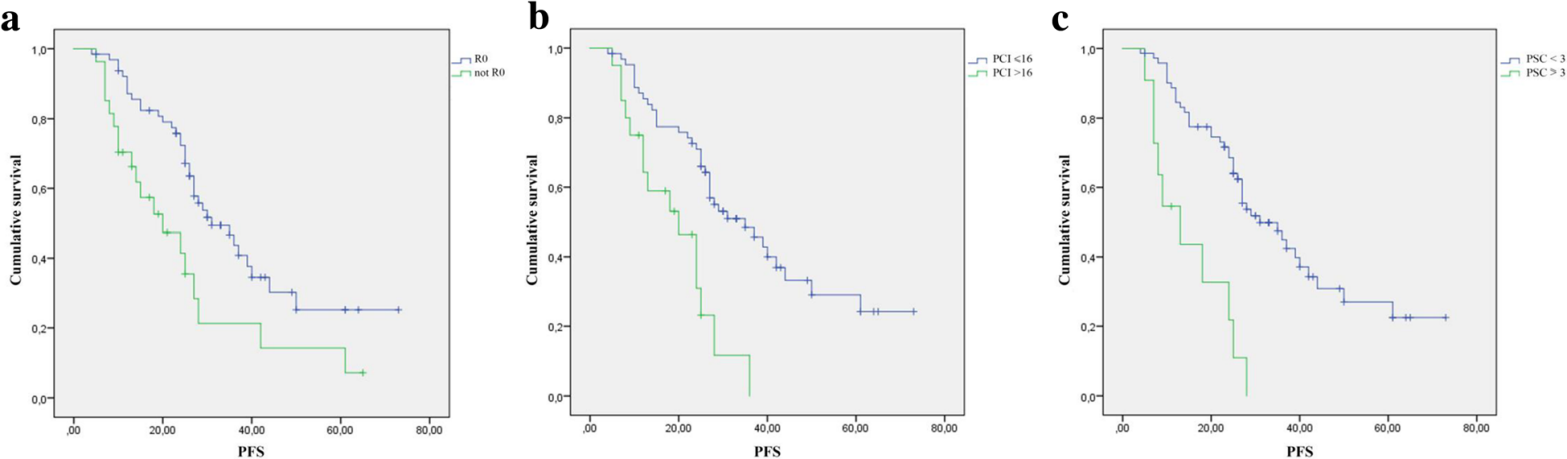
Kaplan-Meyer curves of Progression Free Survival (PFS). **a** PFS according to residual disease at interval debulking surgery (IDS), *R* = 0 vs not; **b** PFS according to Peritoneal Cancer Index (PCI) at IDS, PCI ≤16 vs > 16; **c** PFS according to predictive score of complete cytoreduction (PSC) at IDS, PSC < 3 vs ≥ 3

After a mean follow up of 27 months (range 19.6–34.6 months), 39 patients showed disease progression. Among the variables considered, a PCI > 16 at IDS (*p* < 0.001), a PSC ≥3 (*p* < 0.001) and the presence of residual disease after IDS (*p* = 0.004) were all significantly associated with shorter PFS (Fig. 1).

## Discussion

A key issue in patients with advanced EOC is the selection of patients suitable for complete surgical cytoreduction. Predictive models of surgical outcome based on computed tomography alone [15], or integrated by serum CA-125 levels [21, 22], patient age and performance status [23] have been developed to assist physicians in the decision between PDS or NACT – IDS. Laparoscopic scores have also been proposed [24], with a recent randomized study demonstrating that triage laparoscopy could limit the rate of laparotomies leading to incomplete cytoreduction (“futile laparotomies”) at 10% [25].

If the choice between PDS and NACT-IDS is complex [7], even more controversial is the optimal clinical management of women who undergo NACT according to the indication, timing and extent of IDS based on their stage, comorbidities and, most of all, clinical response [26]. As recently reported, there is still an absence of selecting criteria for patients suitable for NACT/IDS underlining that this approach is still object of debate [27].

Resection of all visible disease should always be the goal in advanced EOC, but it may be particularly important after NACT when patients face their last best chance to receive an effective surgery. Furthermore, selection is crucial, since patients who can be cytoreduced to no macroscopic residual disease may be the only once gaining a survival benefit from surgery at IDS. This opinion is sustained by the randomized study of Vergote et al. where the hazard ratio (HR) of overall survival was not significantly different for *R* = 0 at IDS (HR 1.11; *p* = 0.561) or *R* ≤ 1 cm at PDS (HR 1.37; *p* = 0.130) as compared to R = 0 at PDS (reference), but was significantly worse for R ≤ 1 cm at IDS (HR 1.73; *p* = .0054) [8]. The randomized phase III trial TRUST (NCT02828618) is investigating the role of PDS versus NACT+IDS in large volume comprehensive cancer centers and has already enrolled one third of 686 estimated patients; final results are expected for 2019.

Computed tomography, serum markers and staging laparoscopy have all been investigated for the prediction of complete resection at IDS with variable results. Evaluation of response to NACT by computed tomography is challenging [28] and serum CA-125 variations [29–31] or thresholds [32, 33] have limited accuracy. It has been reported that a laparoscopic score could identify all patients likely to be optimally debulked at IDS, but with the drawback of 32.6% futile laparotomies [34].

At our Institutions, patients with clinical/radiological progressive disease during the first 3–4 courses of NACT, either underwent diagnostic laparoscopy or withdrew chemotherapy, were excluded from the current study. In patients with radiological response/stable disease to NACT we rely on laparotomy to define if radical surgery is appropriate or not. In fact, direct visualization and palpation of the whole abdominal cavity is essential for accurate PCI estimation, which is in turn correlated with tumor resectability and prognosis [20, 35]. Although we acknowledge that the role of PCI in ovarian cancer is under discussion [36, 37] due to its assessment, its low reproducibility and limited utilization, in our series PCI outperformed all other significant predictors and a cut-off < 16 was able to identify almost 90% of patients who could be completely debulked. Nevertheless, this high PPV was obtained with the drawback of 28,6% of unexplored laparotomies. Therefore, we assessed whether other information could add predictive value to PCI by modeling a PSC. Four significant variables reflecting patient (age) and tumor characteristics (PCI and preoperative Ca 125), as well as response to NACT (CA 125 decrease) has been used. Our results indicate that, with a cut a cut-off set at > 4, our PSC may allow to identify all patients who cannot be completely cytoreduced at the price of 15% of futile laparotomies.

In our series, *R* = 0 after IDS was the only parameter significantly associated with PFS. Conversely, in a recent retrospective series from the Mayo Clinic, older age (HR 1.60 per 10-years increase in age) and elevated CA-125 before IDS (HR 2.30 for CA-125 > 35 U/mL) were negatively correlated with OS, while residual disease after IDS did not reach statistical significance (median OS 1.9 vs. 2.6 years; *P* = 0.08) [38]. Indeed, some studies suggest that the degree of pathological response to chemotherapy could be more closely correlated to OS than the absence of residual tumor at IDS [39–42]. Although only *R* = 0 reached statistical significance, the limited number of events of our study may have hindered associations between PFS and other variables, such as age and CA 125 decrease after NACT.

## Conclusions

In conclusion, we showed that our PSC might help surgeons to give a surgical chance to all patients that could be completely debulked, therefore limiting the number of suboptimal surgeries at 16.5%. Both patient’s and tumor’s characteristics likely concur to determine the chance of complete debulking at IDS. Although the influence of tumor chemosensitivity on survival may supersede the once of surgery, the selection of those patients who can be cytoreduced to R = 0 after NACT is crucial to derive the best trade-off from the benefits and the risks of an extensive surgical effort. Our preliminary results suggest that IDS after NACT should be performed in patients with a PSC up to 2, while the value of surgery in patients scoring 4 is likely minimal. In our analysis, we provide a two high-volume-centers experience with standardized multidisciplinary care of EOC. The extrapolation equivalence of PDS and NACT-IDS from the results of randomized studies [8, 9], has been questioned due to patients selection and their poor surgical quality, which led to low both cytoreduction and survival rates [43]. At IDS we obtained a 69% complete cytoreduction rate by performing operations characterized by high surgical complexity, that were guided by the same objective and performed with the same effort as PDS. A prospective validation of the PSC has been already planned at our Institutions.

## Abbreviations

CA-125: cancer antigen-125
CDS: Chronic Disease Score.
CT: Computed tomography.
ECOG: Eastern Cooperative Oncology Group (ECOG).
EOC: epithelial ovarian cancer
FIGO: International Federation of Gynecology and Obstetric
HR: Hazard ratio
IDS: interval debulking surgery
NACT: neodjuvant chemotherapy
PCI: peritoneal cancer index
PDS: Primary debulking surgery.
PFS: progression free survival
PS: performance status
PSC: predictive score of cytoreduction
R: Residual tumor
ROC: Receiver Operating Curve analysis
